# Changes in Thyroid Metabolites after Liothyronine Administration: A Secondary Analysis of Two Clinical Trials That Incorporated Pharmacokinetic Data

**DOI:** 10.3390/metabo12060476

**Published:** 2022-05-24

**Authors:** Nour Diab, Sameer Desale, Mark Danielsen, Josef Köhrle, Nawar Shara, Jacqueline Jonklaas

**Affiliations:** 1 Marshall High School, Falls Church, VA 22043, USA; nouramour3@gmail.com; 2 Center of Biostatistics, Informatics and Data Science, MedStar Health Research Institute, Hyattsville, MD 20782, USA; sameer.desale@medstar.net (S.D.); nawar.shara@medstar.net (N.S.); 3 Department of Biochemistry and Molecular Biology, Georgetown University, Washington, DC 20007, USA; dan@georgetown.edu; 4 Institut für Experimentelle Endokrinologie, Charité–Universitätsmedizin Berlin, Corporate Member of Freie Universität Berlin, Humboldt-Universität zu Berlin, D-10115 Berlin, Germany; josef.koehrle@charite.de; 5 Department of Medicine, Division of Endocrinology, Georgetown University, Washington, DC 20007, USA

**Keywords:** thyroid metabolites, 3,5-diiodothyronine, 3-iodothyronamine, hypothyroidism, liothyronine

## Abstract

We examined relationships between thyroid hormone (TH) metabolites in humans by measuring 3,5-diiodothyronine (3,5-T2) and 3-iodothyronamine (3-T1AM) levels after liothyronine administration. In secondary analyses, we measured 3,5-T2 and 3-T1AM concentrations in stored samples from two clinical trials. In 12 healthy volunteers, THs and metabolites were documented for 96 h after a single dose of 50 mcg liothyronine. In 18 patients treated for hypothyroidism, levothyroxine therapy was replaced by daily dosing of 30–45 mcg liothyronine. Analytes were measured prior to the administration of liothyronine weekly for 6 weeks, and then hourly for 8 h after the last liothyronine dose of the study. In the weekly samples from the hypothyroid patients, 3,5-T2 was higher by 0.033 nmol/L with each mcg/dL increase in T4 and 0.24 nmol/L higher with each ng/dL increase in FT4 (*p*-values = 0.007, 0.0365). In hourly samples after the last study dose of liothyronine, patients with T3 values higher by one ng/dL had 3-T1AM values that were lower by 0.004 nmol/L (*p*-value = 0.0473); patients with 3,5-T2 higher by one nmol/L had 3-T1AM values higher by 2.45 nmol/L (*p*-value = 0.0044). The positive correlations between weekly trough levels of 3,5-T2 and T4/FT4 during liothyronine therapy may provide insight into 3,5-T2 production, possibly supporting some production of 3,5-T2 from endogenous T4, but not from exogenous liothyronine. In hourly sampling after liothyronine administration, the negative correlation between T3 levels and 3-T1AM, but positive correlation between 3,5-T2 levels and 3-T1AM could support the hypothesis that 3-T1AM production occurs via 3,5-T2 with negative regulation by T3.

## 1. Introduction

Hypothyroidism is treated with the precursor thyroxine (T4), and sometimes with its metabolite triiodothyronine (T3) using levothyroxine (LT4) and liothyronine (LT3), respectively. The impact of LT4 is exerted when it is converted from the prohormone T4 to the active hormone T3. T3 binds to its receptor, enabling binding to the thyroid response element, and thereby initiating the modulation of T3-responsive genes and the tissue impact of thyroid hormone (TH) [1]. Although the production of the inactive metabolite reverse T3 is well elucidated, the role of other metabolites of T4 and T3 such as 3,5-diiodothyronine (3,5-T2), and 3-iodothyronamine (3-T1AM) in potentially mediating the effects of TH and the pathways by which they are produced are not fully understood [2,3]. 3,5-T2 has thyromimetic actions when administered in larger doses in rodents, while causing stimulation of oxygen consumption and lipid metabolism in lower doses, likely not via interaction with the thyroid hormone receptor [4,5]. 3-T1AM causes increased lipid metabolism, reduced fat mass, lowered body temperature and may also have stimulatory effects on memory in animal models and anti-proliferative effects on cancer cells in vitro [2,3,5]. The pharmacologic actions of these agents remain to be dissected from their physiologic effects.

3,5-T2 may be produced from T3 by via the actions of deiodinases. In keeping with this theory, 3,5-T2 concentrations have been reported to be higher in LT4-treated thyroidectomized patients than in euthyroid individuals [6]. It has also been suggested that 3-T1AM may be produced in extrathyroidal tissues from LT4 due to its elevated levels in patients with thyroid cancer treated with LT4, compared with matched healthy controls [7]. However, the responsible enzyme does not appear to be aromatic L-amino acid decarboxylase, but may be another decarboxylase [7]. Interestingly, 3,5-T2 serum concentrations did not differ between hyperthyroid compared to hypothyroid individuals [6]. A recent study used tandem mass spectrometry to measure several thyroid hormone metabolites and found levels to be higher in thyroidectomized patients receiving TSH suppression therapy [8]. However, 3,5-T2 levels were below the detection limit of the assay.

In this secondary analysis, we utilized stored samples from two previous studies of LT3 administration [9,10]. The first study involved a single dose of LT3 given to healthy euthyroid volunteers and provided LT3 pharmacokinetic parameters [9]. The second study involved patients with hypothyroidism predominately due to Hashimoto’s thyroiditis who were switched from LT4 therapy to daily LT3 for 6 weeks. Significant excursions in T3 levels were documented but there was no change in clinical parameters or biomarkers of thyroid hormone action [10]. Secondary analysis of the TH metabolites (3,5-T2 and 3-T1AM) levels from these studies was performed to assess their relationship with T4, free T4 and T3. The rationale for this study was to determine if data from participants with complete or partial endogenous thyroid function receiving LT3 may complement the data from thyroidectomized individuals treated with LT4 [6,7,8] and shed light on the pathways for production of 3,5-T2 and 3-T1AM. It was hoped that these preliminary data would be useful to generate hypotheses that could subsequently be investigated in future hypothesis-testing studies.

## 2. Results

**Figure 1:**
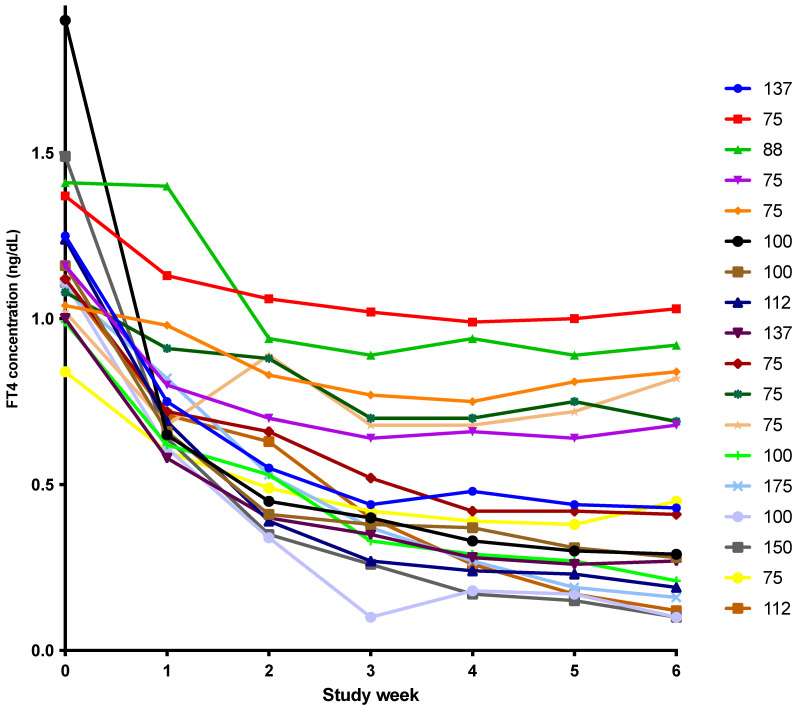
Weekly drop in FT4 concentration with LT3 therapy in 18 patients with hypothyroidism. (FT4: free thyroxine, LT3: liothyronine. The legend on the right of the figure indicates the LT4 dose being taken by each patient prior to the start of the study.)

A preliminary version of these results was presented 1 October 2021 as a virtual abstract at the 90th Annual Meeting of the American Thyroid Association (Poster #178). Study data is available in the Supplemental Materials. For the first study, 12 healthy participants were eligible and completed the study (Detailed description provided in [9]). The participants were 4/12 (33%) women, 7/12 (58%) Caucasian, 5/12 (42%) African American and had a mean age of 29 years (age range 18–43 years). They completed the study in 2012. For the second study [10], 18 patients were eligible and completed the study. They were 16/18 (89%) women, 13/18 (72%) Caucasian, 3/18 (17%) African American, 2/18 (11%) Asian or Hispanic and had a mean age of 38 years (age range 24–56 years). The mean dose of LT4 at study entry was 102 mcg. However, the doses of LT4 varied from 75 mcg to 175 mcg and as can be shown from the decline in FT4 concentrations after LT4 was discontinued and LT3 monotherapy was provided, this correlated with varying nadirs in FT4 concentration (see Figure 1), presumably reflecting different degrees of residual endogenous thyroid function. The study was completed between April 2013 and July 2014. The analysis of the TSH, T4, FT4, T3 and FT3 levels and the pharmacokinetics from these studies has been reported previously [9,10].

### 2.1. Healthy Volunteers Receiving a Single Dose of LT3

#### 2.1.1. Trends over Time

**Figure 2:**
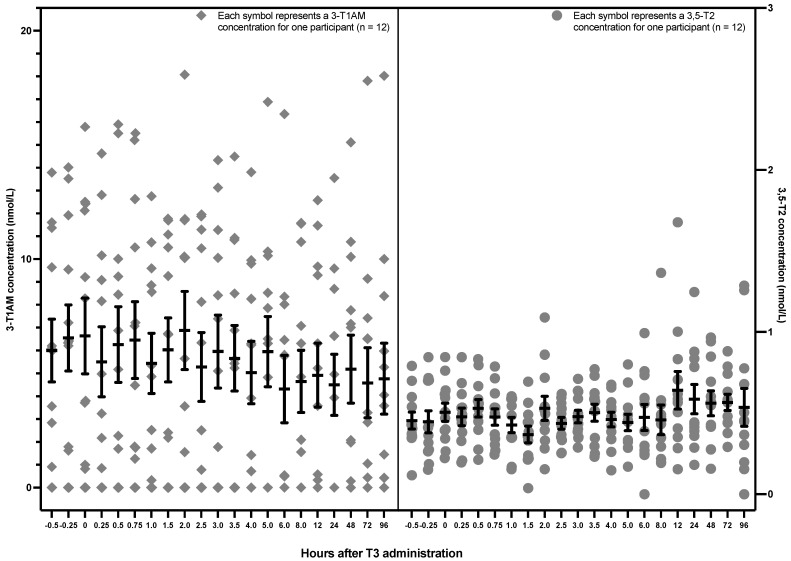
Hourly 3,5-T2 and 3-T1AM concentrations after a single 50 mcg dose of LT3 in 12 healthy volunteers (The lower limit of detection is 0.2 nmol/L for 3,5-T2 and approximately 5 nmol/L for 3-T1AM. 3,5-T2: 3,5-diiodothyronine, 3-T1AM: 3-iodothyronamine, LT3: liothyronine).

**Figure A1:**
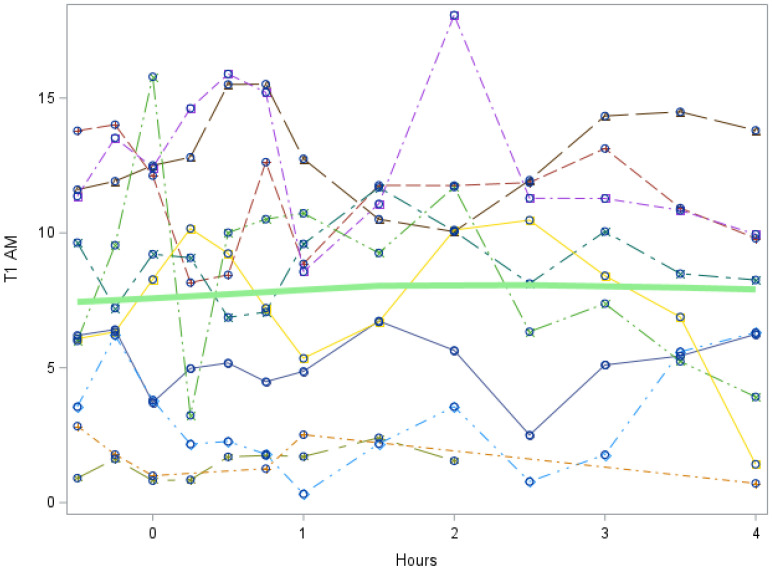
**Study 1:** 3-T1AM trend in first 5 h after LT3 dose (Each individual line represents a participant. The green line represents the mean values for all participants). 3-T1AM = 3-T1AM concentration in nmol/L. Slope of line = −0.14, *p* value = 0.3687. This indicates that there is no significant trend in 3-T1AM during the first 5 h.

**Figure A2:**
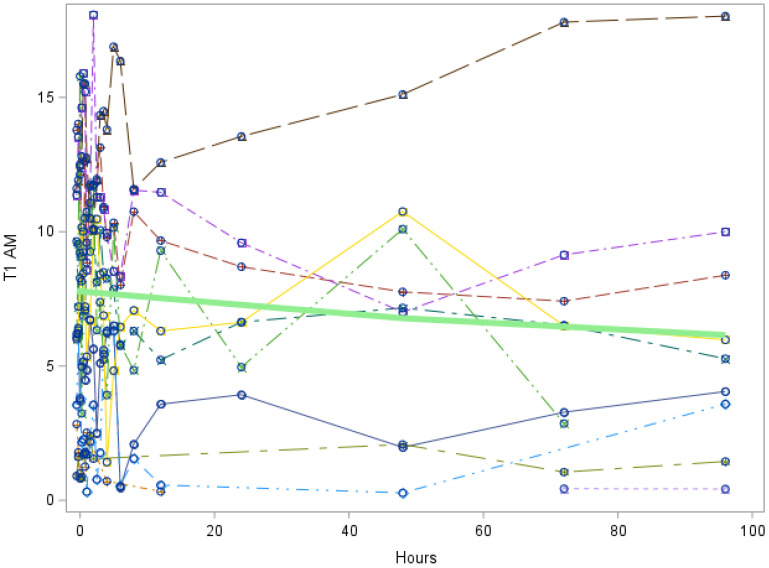
**Study 1:** 3-T1AM trend over all hours after LT3 dose (Each individual line represents a participant. The green line represents the mean values for all participants). 3-T1AM = 3-T1AM concentration in nmol/L. Slope of line = −0.012, *p* value = 0.1859. This indicates that there is no significant trend in 3-T1AM during the study duration.

**Figure A3:**
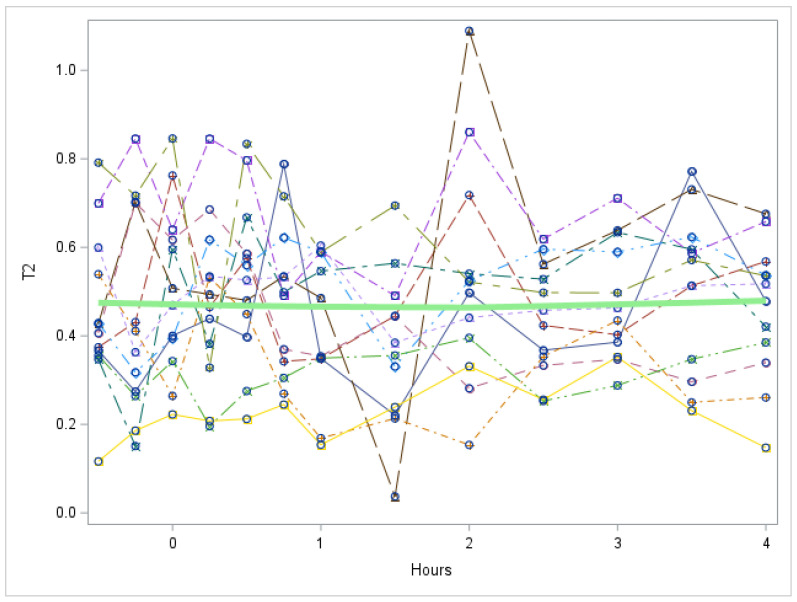
**Study 1:** 3,5-T2 trend in first 5 h after LT3 dose (Each individual line represents a participant. The green line represents the mean values for all participants). T2 = 3,5-T2 concentration in nmol/L. There is no significant change in 3,5-T2 during the first five hours, *p* value = 0.7421.

**Figure A4:**
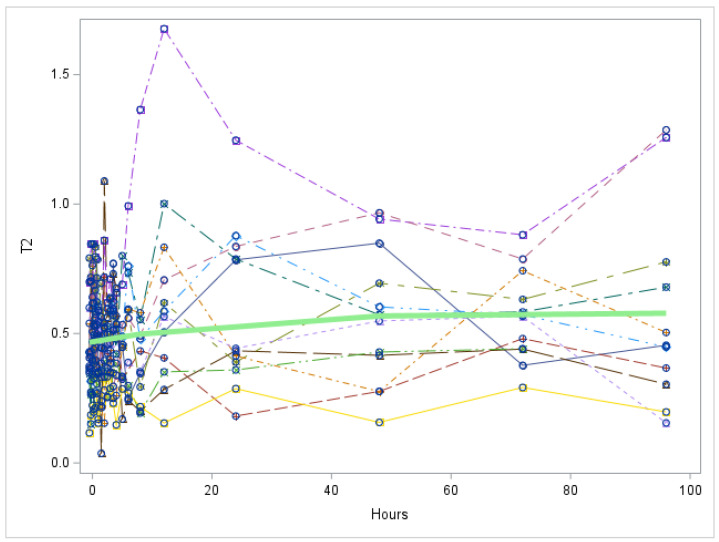
**Study 1:** 3,5-T2 trend over all hours after LT3 dose (Each individual line represents a participant. The green line represents the mean values for all participants). T2 = 3,5-T2 concentration in nmol/L. There is no significant change in 3,5-T2 over time, *p* value = 0.1901.

The 3,5-T2 and 3-T1AM concentrations are displayed based on the hourly sampling time points in Figure 2. 3,5-T2 and 3-T1AM levels remained stable after the administration of 50 mcg LT3. There was also no trend for a change in 3,5-T2, either over the first 5 h (*p* value = 0.75) or over all 96 h (*p* value = 0.19). Similarly, there was no change in 3-T1AM either over the first 5 h (*p* value = 0.3687) or over all 96 h (*p* value = 0.1859) (see Table 1). Data are displayed for each participant in Appendix A (Figure A1, Figure A2, Figure A3 and Figure A4).

#### 2.1.2. TH and TH Metabolite Interrelationships

There was also no correlation between 3,5-T2 and 3-T1AM concentrations (see Table 2). Finally, there was no relationship between TSH, T4, FT4, T3 and FT3 levels and the observed 3,5-T2 and 3-T1AM concentrations (see Appendix B Table A1 and Table A2).

#### 2.1.3. Participant Characteristics

**Figure 3:**
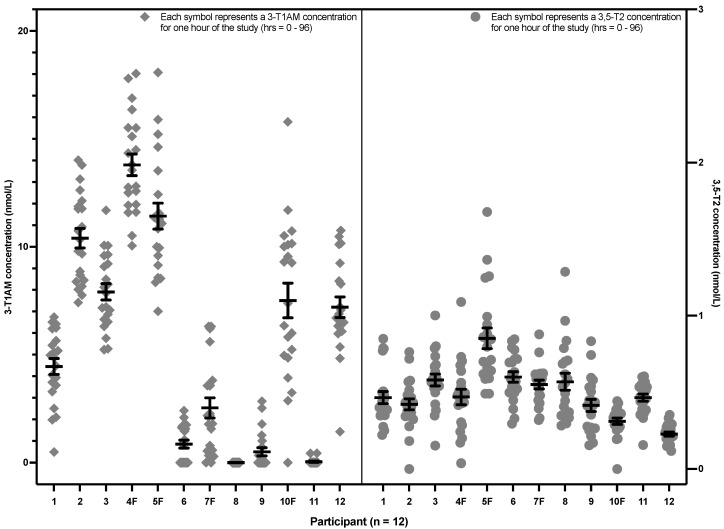
3,5-T2 and 3-T1AM concentrations from the hourly sampling after a single 50 mcg dose of LT3 grouped by the 12 individual healthy volunteers (The lower limit of detection is 0.2 nmol/L for 3,5-T2 and approximately 5 nmol/L for 3-T1AM. 3,5-T2: 3,5-diiodothyronine, 3-T1AM: 3-iodothyronamine, LT3: liothyronine).

Figure 3 shows the same data, in contrast grouped according to each participant, rather than by time point. It can be seen that each participant had an apparent setpoint for their 3,5-T2 and 3-T1AM concentrations. There was no effect of sex, race, age and BMI on 3,5-T2 concentrations (see Appendix B Table A1). For 3-T1AM, there was also no effect of race, age and BMI. However, females (indicated by the letter F after their participant number in Figure 3) had a 3.9-fold higher concentration of 3-T1AM when compared to the levels of the male participants (*p* value = 0.034) (see Appendix B Table A2).

### 2.2. Patients with Hypothyroidism Receiving a Daily Dose of LT3

#### 2.2.1. Trends over Time

**Figure 4:**
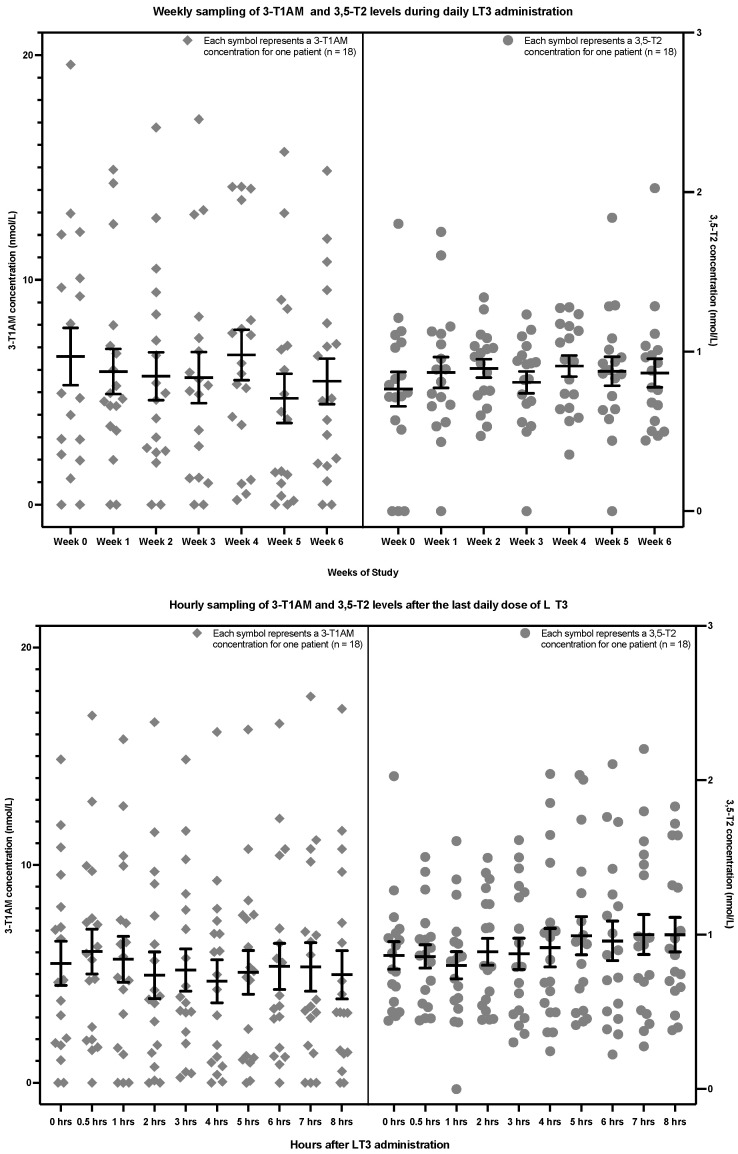
Weekly 3,5-T2 and 3-T1AM concentrations at the time of trough T3 concentrations (upper section) and hourly 3,5-T2 and 3-T1AM concentrations after the final LT3 dose of the study (lower section) in 18 patients with hypothyroidism (The lower limit of detection is 0.2 nmol/L for 3,5-T2 and approximately 5 nmol/L for 3-T1AM. 3,5-T2: 3,5-diiodothyronine, 3-T1AM: 3-iodothyronamine, LT3: liothyronine).

**Figure A5:**
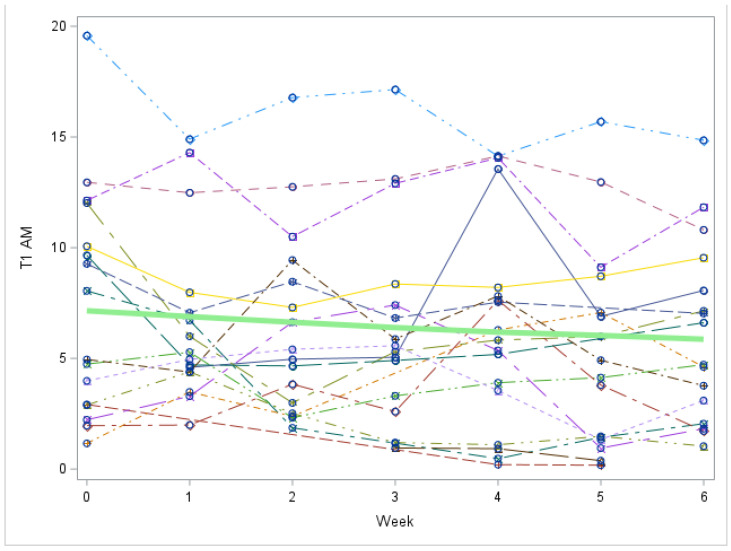
**Study 2 by Week:** 3-T1AM trend over time (Each individual line represents a patient. The green line represents the mean values for all patients). 3-T1AM = 3-T1AM concentration in nmol/L. There is no significant change in 3-T1AM over time, *p* =0.3633.

**Figure A6:**
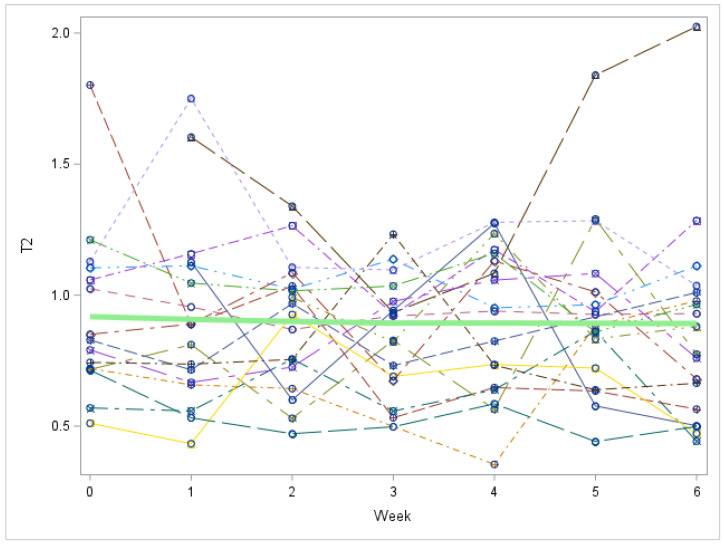
**Study 2 by Week:** 3,5-T2 trend over time (Each individual line represents a patient. The green line represents the mean values for all patients). T2 = 3,5-T2 concentration in nmol/L. There is no significant change in 3,5-T2 over time, *p* = 0.4982.

**Figure A7:**
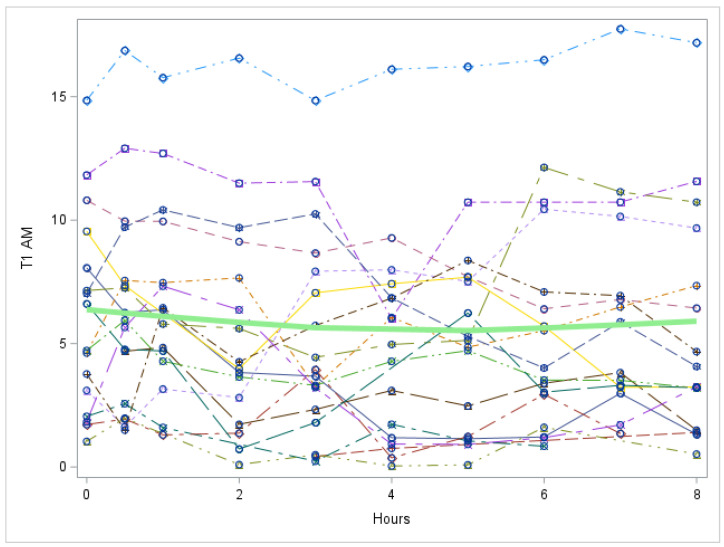
**Study 2:** 3-T1AM trend over time after last LT3 dose of the study (Each individual line represents a patient. The green line represents the mean values for all patients)**.** 3-T1AM = 3-T1AM concentration in nmol/L. There is no significant trend in 3-T1AM over time, *p* value = 0.4177.

**Figure A8:**
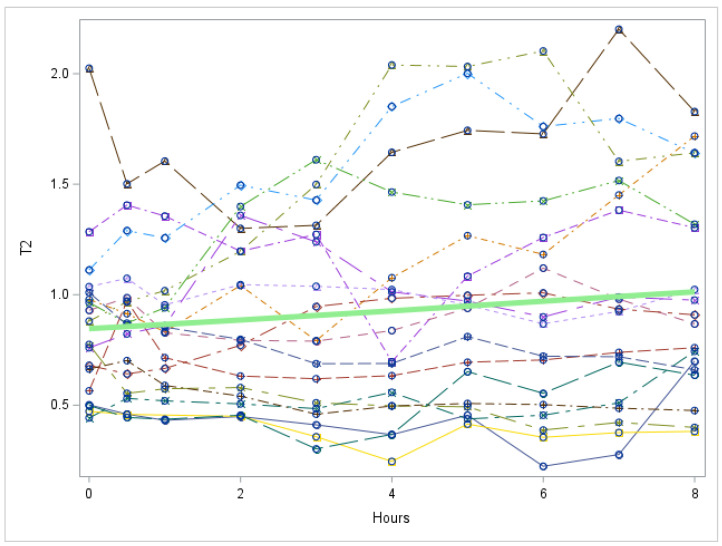
**Study 2:** 3,5-T2 trend over time after last LT3 dose of the study (Each individual line represents a patient. The green line represents the mean values for all patients). T2 = 3,5-T2 concentration in nmol/L. There is no significant change in 3,5-T2 over time, *p* value = 0.1256.

In the second study of the patients with hypothyroidism, the upper section of Figure 4 shows the weekly sampling of 3,5-T2 and 3-T1AM levels associated with trough levels of T3, while the lower section of Figure 4 shows the hourly sampling after the final dose of daily LT3 of the study. There was no significant trend in 3,5-T2 levels in either the weekly (*p* = 0.4982) or hourly sampling (*p* value = 0.1256). Similarly, there was also no significant trend in 3-T1AM levels in either the weekly (*p* = 0.3633) or hourly sampling (*p* value = 0.4177) (see Table 3). Data are displayed for each patient in Appendix A (Figure A5, Figure A6, Figure A7 and Figure A8).

#### 2.2.2. TH and TH Metabolite Interrelationships

During the weekly sampling of the hypothyroid patients treated with daily LT3, there were no significant correlations between 3,5-T2 and 3-T1AM levels (*p* value = 0.1457 without using an interaction term between 3,5-T2 and time and *p* value = 0.9785 using an interaction term between 3,5-T2 and time) (see Table 4). However, for the hourly sampling after the last dose of LT3, 3,5-T2 and 3-T1AM were positively associated, with higher 3,5-T2 levels being associated with higher 3-T1AM levels. Patients with 1 nmol/L higher 3,5-T2 levels on average had 3-T1AM levels that were higher by 2.45 nmol/L (*p* value = 0.0041) (see Table 5). This correlation between 3,5-T2 and 3-T1AM did not change over time.

The data from the weekly sampling showed that T4 and free T4 concentrations were directly associated with 3,5-T2. 3,5-T2 levels increased by about 0.033 nmol/L per each mcg/dL increase in T4 (*p* value = 0.007) and 3,5-T2 also increased by about 0.24 nmol/L per each ng/dL increase in free T4 (*p* value = 0.03). There were no associations between 3-T1AM and TSH, T4, FT4, T3 and FT3 in the weekly samples. In the hourly samples taken after the last dose of LT3, there were no associations between 3,5-T2 and TSH, T4, FT4, T3 and FT3. However, T3 levels and 3-T1AM levels were negatively correlated. For each ng/dL increase in T3, 3-T1AM levels were lower by 0.004 nmol/L (*p* value = 0.047).

#### 2.2.3. Patient Characteristics

**Figure 5:**
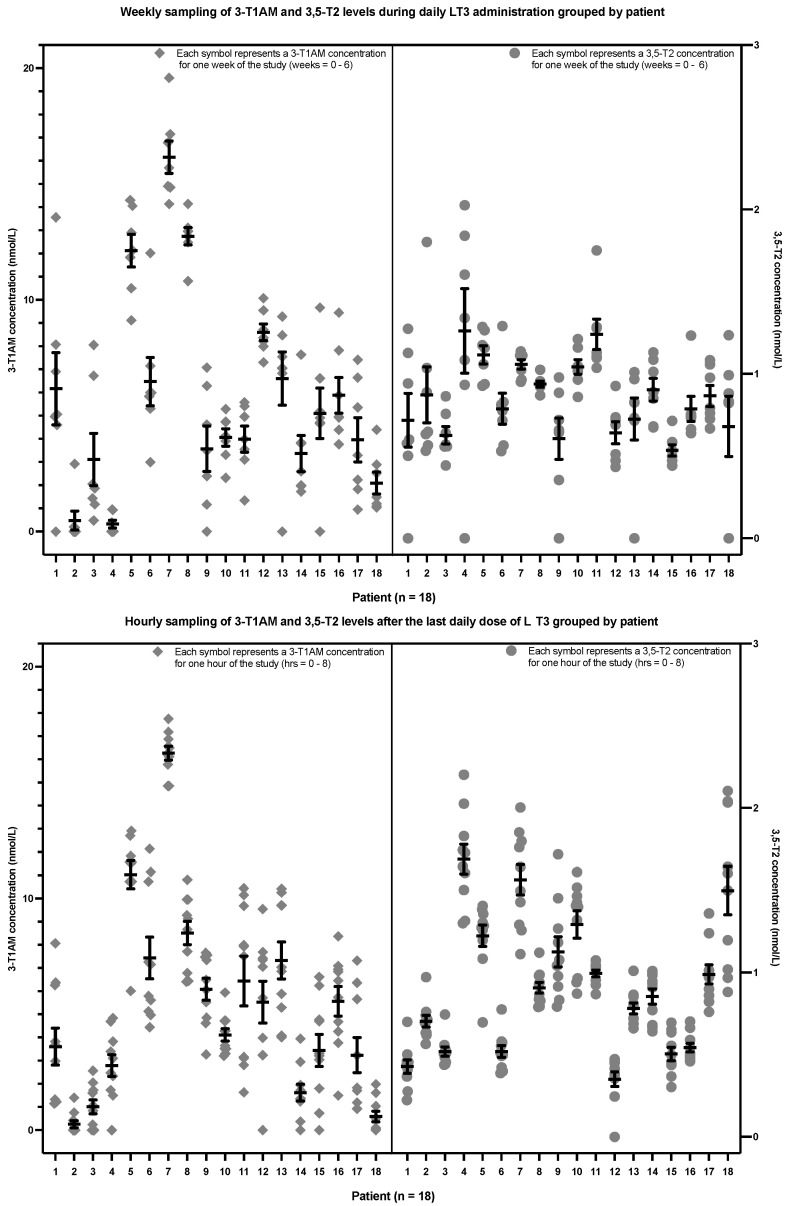
3,5-T2 and 3-T1AM concentrations from the weekly (upper section) and hourly sampling (lower section) grouped by the 18 individual patients with hypothyroidism (The lower limit of detection is 0.2 nmol/L for 3,5-T2 and approximately 5 nmol/L for 3-T1AM. 3,5-T2: 3,5-diiodothyronine, 3-T1AM: 3-iodothyronamine, LT3: liothyronine).

Figure 5 shows the same data from the participants with hypothyroidism. However, rather than showing the data grouped according to time (either weekly or hourly as in Figure 4), they are shown grouped by each of the 18 patients. The upper section of Figure 5 shows the data from the weekly sampling, whereas the lower section of Figure 5 shows the data from the hourly sampling. It can be seen that each participant had an apparent setpoint for their 3,5-T2 and 3-T1AM concentrations. There was no effect of sex, race, age and BMI on 3,5-T2 or 3-T1AM concentrations (see Appendix B Table A3, Table A4, Table A5 and Table A6).

## 3. Discussion

With regard to our first research question of whether there were within individual changes in 3-T1AM and 3,5-T2 across the time points of the two studies, we did not identify such changes. This was true for both the healthy volunteers given a single dose of LT3 and the patients treated with daily LT3. The lack of trends over time in 3,5-T2 and 3-T1AM during the successive weeks, as LT4 therapy was replaced with LT3 therapy in the hypothyroid patients, suggests that 3,5-T2 and 3-T1AM, regardless of the source of T3 (direct or from LT4) and the varied amount of residual thyroid function as a source of T4 in the participants, is protected and maintained relatively constant. This was true not only for the weekly trough levels, which might be expected to be more constant, but even acutely during hourly sampling after LT3 administration. These data do not provide support for the suggestion that 3,5-T2 is produced from exogenous T3. However, these data suggest that 3,5-T2 and 3-T1AM levels are defended within particular limits.

Our second research question probed relationships between 3,5-T2 and 3-T1AM and queried whether there were paired, linked or reciprocal changes. No relationship was observed between 3,5-T2 and 3-T1AM levels during the weekly sampling in the hypothyroid patients, suggesting the tight regulation of metabolites. However, during the dynamic sampling after the administration of the last LT3 dose, there was a positive correlation between 3,5-T2 and 3-T1AM which could suggest that 3-T1AM production is via 3,5-T2. Acutely after the T3 rise, it could be hypothesized that 3,5-T2 levels rose and resulted in higher 3-T1AM concentrations. However, such a hypothesis does not explain why higher T3 levels were associated with lower 3-T1AM levels unless T3 negatively regulated 3-T1AM production from 3,5-T2.

We also queried whether there were changes in 3,5-T2 or 3-T1AM that were related to or linked to the corresponding T4, T3, FT4, FT3 and TSH values. The fact that no acute changes in 3,5-T2 or 3-T1AM were observed after exogenous LT3 administration in individuals with intact thyroid function, despite the fact that their mean T3 concentrations rose from 120 ng/dL to 346 ng/dL, either suggests that these metabolites are not produced via exogenous T3 or that metabolite concentrations are tightly and rapidly regulated. The lack of relationships between TH concentrations and TH metabolites concentrations in these same individuals with native thyroid function also suggests rapid autoregulation in individuals with normal endogenous thyroid function. Some possible hypotheses for the stability of 3,5-T2 or 3-T1AM might include tight binding to ApoB100. The possibility that 3-T1AM might be tightly bound to ApoB100 has been suggested previously [11], but there are no comparable data on 3,5-T2. Both 3,5-T2 and 3-T1AM might be preferably intracellularly generated and localized in specific tissues (such as the liver), with these metabolites then slowly “leaking” from such sites into the circulation.

During the weekly steady state sampling in the hypothyroid patients, a positive correlation between both T4 and free T4 levels and 3,5-T2 concentrations was observed, which could suggest that 3,5-T2 is produced from T4. Other studies have suggested extra-thyroidal production of TH metabolites from exogenous LT4 in athyreotic individuals [6,7,8]. However, the hypothyroid patients in our second study retained at least some degree of residual endogenous thyroid function and were exclusively receiving LT3. They would have had some remaining T4 from exogenous LT4 at the beginning of the study, which then declined, so that by the end of the study any T4 would be from endogenous sources. This could raise the possibility that 3,5-T2 can also be made from endogenous T4, but does not support the concept of 3,5-T2 production from exogenous T3.

Our final research question was whether 3,5-T2 or 3-T1AM values, or changes in 3,5-T2 and 3-T1AM values, were related to age, sex, or other characteristics such as BMI. We did not find any relationships between these characteristics and TH metabolites, with one exception. Our finding of a sex difference in 3-T1AM concentrations in those with endogenous normal thyroid function is a novel finding that has yet to be confirmed. Given the small number of individuals studied, this may not be a true finding. If it is confirmed in future adequately powered studies that TIAM levels are indeed higher in women, this could potentially be due to higher thyroxine-binding globulin levels in women, direct stimulation from sex-specific steroid hormones or other unknown factors. However, no sex difference in 3-T1AM levels was observed in the treated hypothyroid patients in the study, and, admittedly, this theory does not explain why these findings were not also seen in women being treated for hypothyroidism.

However, interestingly, we did find that both healthy volunteers and hypothyroid patients seemed to have personal ranges for 3,5-T2 and 3-T1AM. The distribution of the data points for 3,5-T2 and 3-T1AM from individuals with intact thyroid function suggests that there is a distinct setpoint for these metabolites for each individual participant that is composed of closely grouped values and is not subject to perturbations caused by exogenous LT3. Similar individual setpoints have been observed for TSH, T4 and T3 [12,13]. These setpoints also seem to extend to LT4-treated patients [14,15,16]. As was the case in the euthyroid individuals, the data from the individuals with treated hypothyroidism also suggested characteristic individual setpoints for TH metabolites.

There is a rich literature describing patient dissatisfaction with LT4 therapy [17,18] and patient preferences for synthetic combination therapy and desiccated thyroid extract [1,19,20]. There has been speculation that the levels of TH metabolites achieved during LT4 monotherapy may provide some underpinning for this dissatisfaction [2,8]. Our data do not support this theory as TH metabolites did not appear to change during the transition from LT4 to LT3. In addition, our study was uncontrolled and so could not assess satisfaction with therapy during the transition. A prior study of TH metabolites has also reported that there was no association between 3,5-T2 concentrations and quality of life in patients with thyroid cancer who were receiving LT4 replacement [21].

The strengths of our studies are that we are able to provide some data from volunteers and patients with normal and partial endogenous thyroid function, respectively, receiving LT3, thus potentially complementing data regarding athyreotic LT4-treated patients already reported in the literature [6,7,8]. We were also able to measure not only 3,5-T2 concentrations, but 3-T1AM concentrations too, which have not been included in recent studies utilizing tandem mass spectrometry [8]. The limitations of our study are numerous. Foremost, we did not utilize the very specific tandem mass spectrometry methodology for measuring TH metabolites. Our samples had been stored for several years prior to their use for this secondary analysis. However, the samples were appropriately stored in a −80° centigrade freezer with continuous monitoring, thus ensuring sample quality. There were no intervening episodes of thawing prior to use. Additionally, our participant numbers were very small and this was a secondary analysis of two prior studies with different designs performed for other purposes. Due to the multiple comparisons in the studies, the authors cannot exclude the possibility of multiple testing bias. The sex and age composition of the participants in the two studies differed. In addition, the inclusion of individuals with varying degrees of endogenous thyroid function (full endogenous function in study one and variable degrees of residual function in study two) added complexity to separating the influences of endogenous versus exogeneous sources of T4. An additional layer of complexity was that the hypothyroid participants in study two were being transitioned off LT4 therapy to LT3 therapy. Although the limitations of this secondary analysis make hypothesis testing challenging, these data may be useful for generating hypotheses to test in future studies.

There are currently challenges and controversies in the field of clinical chemistry regarding the measurement of the serum concentrations of 3,5-T2 and 3-T1AM [22,23]. There are differences within the groups who have developed mass spectrometry assays [22] and immunoassay-based data do not correlate with mass spectrometry data. For example, 3,5-T2 is below the limit of detection using mass spectrometry assays [8,24], compared with levels of 0.253 ± 0.029 nmol/L detected in healthy individuals using immunoassay [6]. Another mass spectrometry method found average 3,5-T2 concentration of 78 ± 9 pmol/L in euthyroid individuals [25]. Such differences are presumably due to various methodologic and technologic considerations, including those pertaining to internal standards. Some mass spectrometry assays have not detected 3-T1AM [8,22]. In one case due to sample preparation being selective for negatively charged ions, as opposed to positively charged ones as would be present in 3-T1AM [8]. Comparing studies reporting the measurement of 3-T1AM in human samples, these studies reported average concentrations varying between 0.219 nmol/L measured by mass spectrometry [26] and 14–66 nmol/L measured by immunoassay [7,27]. Determination of TH metabolites from human samples simultaneously measured by immunoassay and tandem mass spectrometry would be helpful.

To summarize our findings, the positive correlations between weekly trough levels of 3,5-T2 and both T4 and FT4 during T3 therapy and their significance with respect to 3,5-T2 production is unclear. However, this finding may possibly suggest some production of 3,5-T2 from endogenous T4, but not from exogenous T3. In the acute hourly sampling immediately after T3 administration the negative correlation between T3 levels and 3-T1AM, but positive correlation between 3,5-T2 and 3-T1AM could lead to speculation that 3-T1AM production is from 3,5-T2 with negative regulation by T3. However, more studies are needed both to elaborate the pathways for TH metabolite production in the native euthyroid versus the hypothyroid LT3-replaced state versus the hypothyroid LT4-replaced state, and to clarify the pathways for 3-T1AM production. During LT3 treatment, both endogenous euthyroid volunteers and hypothyroid patients seem to maintain individual setpoints for their TH metabolite concentration.

## 4. Materials and Methods

This report involves a secondary analysis of additional laboratory testing performed on stored samples from two prior studies [9,10]. Participants in both studies provided written informed consent, which included permission for future testing of stored samples. TH metabolite measurement was not pre-planned at the time of the initial studies and a formal power analysis was not performed. The research questions considered in these analyses were: (a) are there within individual changes in 3-T1AM and 3,5-T2 across the time points of the 2 studies, (b) are there relationships between any 3,5-T2 and 3-T1AM changes (paired, linked or reciprocal changes, etc.), (c) are there changes in 3,5-T2 or 3-T1AM that are related to or linked to the corresponding T4, T3, FT4, FT3 and TSH values and (d) are the 3,5-T2 or 3-T1AM values, or changes in 3,5-T2 and 3-T1AM values, related to individual age, sex or other characteristics?

The first study involved healthy volunteers with normal thyroid function who were given a single dose of 50 mcg LT3. The trial was approved by the Institutional Review Board and registered at ClinicalTrials.gov as clinical trial NCT01581463 (Detailed description provided in [9]). Volunteers aged 18–55 years without thyroid disease were recruited and screened by telephone for any conditions or medications known to affect thyroid hormone (TH) metabolism, absorption or binding. Potentially eligible participants were scheduled for an in-person visit to the Georgetown University Clinical Research Unit (GCRU) and signed a written consent form for the study. A medical history, physical examination, electrocardiogram and screening TSH were then obtained. Participants in good health and with a TSH value within the reference range (0.4–4.5 mIU/L) were then scheduled for pharmacokinetic testing. Testing commenced at 7:30 am after an overnight fast. The 50 mcg LT3 dose was administered at 8:00 am. Blood samples were collected at 15 min interval for 4 samples, then at 30 min intervals for 6 samples, hourly for 2 samples and then at 6, 8, 12, 24, 48, 72 and 96 h after LT3 administration. Samples were initially assayed for TSH, T4, FT4, T3 and FT3 at the Georgetown University Laboratory in one batch for each participant using a Siemens Dimension Vista Analyzer [9]. Later, all samples for all participants were measured for 3,5-T2 and 3-T1AM in 2017 at Charité–Universitätsmedizin Berlin [2].

The second group was composed of patients with hypothyroidism in whom their usual LT4 treatment was replaced with daily LT3 for 6 weeks. This study was approved by the Institutional Review Board and registered at ClinicalTrials.gov as clinical trial NCT01800617 (Detailed description provided in [10]). Briefly, participants aged 18–65 years with hypothyroidism of any etiology and taking at least 75 mcg LT4 daily were recruited and screened by telephone to exclude individuals with any significant medical conditions and any conditions or medications known to affect TH metabolism, absorption or binding. Potentially eligible participants were seen on the GCRU where they signed a written informed consent form and underwent a medical history, physical examination, electrocardiogram and screening TSH measurement. Participants with hypothyroidism and otherwise in good health and with a TSH within the reference range (0.4–4.5 mIU/L) while taking LT4 were then studied for 6 weeks. At the initial visit the participant was asked to present after discontinuing their LT4 therapy (last dose of LT4 taken the day prior), baseline pre-LT3 dose (trough) measurements of TSH, T4, FT4, T3 and FT3 were obtained, and the patient was started on 15 mcg LT3 once daily. A week later, a second set of trough (pre-LT3 dose) thyroid function tests was obtained and the participant continued on 15 mcg LT3 daily. The following week after the trough thyroid function tests were obtained the participants were switched to either 30 mcg or 45 mcg LT3 based on their pre-study dose of LT4. There were then 4 subsequent visits at which trough levels of TSH, T4, FT4, T3 and FT3 were obtained. At the time of the seventh and final study visit, following the baseline thyroid function tests, the participants took their final LT3 dose of the study and thyroid function tests were repeated at 30 min and then hourly for eight hours in an abbreviated pharmacokinetic study. All samples were assayed at the Georgetown University Laboratory using a Siemens Dimension Vista Analyzer on the day they were obtained for LT3 dose adjustment and safety monitoring [10].

Samples from both studies were later analyzed for TH metabolites (3,5-T2 and 3-T1AM) in 2017 at Charité–Universitätsmedizin Berlin. The samples from both studies were pooled together and placed in a randomized order prior to analysis to avoid between-run bias. The detailed assay methodology using a monoclonal antibody-based chemiluminescence immunoassay has been previously described [2,6,7] and preliminary analysis of the 3,5-T2 and 3-T1AM concentrations from these cohorts has also been described [2].

### Statistical Methods

Continuous variables were time in hours or time in weeks. A linear regression model was fit to the data to measure the change in 3-T1AM and 3,5-T2 concentrations over time by calculating the slope of a line. Considering the repeated measures within individuals, linear regression was fit using a repeated measures model with autoregressive covariance structure. During the first study, measurements were collected at shorter intervals during the first 5 h and at larger intervals during later hours, hence analysis was conducted over all 96 h as well as for the subset of measurements during the first 5 h.

Correlation between 3-T1AM and 3,5-T2 concentrations was tested using a linear regression model with repeated measures and autoregressive covariance structures. 3-T1AM was considered as a dependent variable and 3,5-T2 as an independent variable. The model also adjusted for time as a continuous variable. An additional model was constructed to include interaction between time and 3,5-T2 to test the change in correlation between 3-T1AM and 3,5-T2 over time. Similarly, the relationship between 3-T1AM or 3,5-T2 with T4, T3, FT3, FT4, TSH and individual characteristics such as age and sex was tested using a linear regression model with repeated measures and autoregressive covariance structure controlling for time as a continuous variable. Analysis was conducted using SAS 9.4. Statistical analysis did not utilize listwise deletion. Missing data were addressed using the “Proc Mixed” procedure in SAS. This analyzes all of the data that are present with the assumption that the data are missing at random.

## 5. Conclusions

These data may have clinical significance through suggesting differential production of metabolites from exogenous levothyroxine versus liothyronine. The positive correlations between weekly trough levels of 3,5-T2 and T4/FT4 during liothyronine therapy may potentially provide insight into 3,5-T2 production. These findings possibly support some production of 3,5-T2 from endogenous T4, but not from exogenous liothyronine. In hourly sampling after liothyronine administration in hypothyroid patients the negative correlation between T3 levels and 3-T1AM, but positive correlation between 3,5-T2 levels and 3-T1AM could support the hypothesis that 3-T1AM production occurs via 3,5-T2 with negative regulation by T3.

## Figures and Tables

**Table 1:** Changes in 3,5-T2 and 3-T1AM over time in the healthy volunteers.

Time Period	Effect	Estimate	Standard Error	DF	*t* Value	*p* Value
3,5-T2 (first 5 h)	Intercept	0.4657	0.02553	11	18.24	<0.0001
	Hour (slope)	−0.00281	0.008537	143	−0.33	0.7421
3,5-T2 (96 h)	Intercept	0.4741	0.02134	11	22.21	<0.0001
	Hour (slope)	0.000792	0.000602	237	1.31	0.1901
3-T1AM (first 5 h)	Intercept	7.0201	0.8195	9	8.57	<0.0001
	Hour (slope)	−0.1355	0.1501	108	−0.90	0.3687
3-T1AM (96 h)	Intercept	6.5070	0.7126	10	9.13	<0.0001
	Hour (slope)	−0.01177	0.008862	174	−1.33	0.1859

**Table 2:** Correlations between 3,5-T2 and 3-T1AM concentrations in the healthy volunteers. (* = interaction term).

**Correlation between 3,5-T2 and 3-T1AM (Model without Using an Interaction Term between 3,5-T2 and Time)**					
**Effect**	**Estimate**	**SE**	**DF**	***t* Value**	***p* Value**
Intercept	7.0819	1.0007	10	7.08	<0.0001
3,5-T2	0.1785	1.0375	172	0.17	0.8636
t	−0.01344	0.009318	172	−1.44	0.1511
**Correlation between 3,5-T2 and 3-T1AM (Model Using an Interaction Term between 3,5-T2 and Time)**					
**Effect**	**Estimate**	**SE**	**DF**	***t* Value**	***p* Value**
Intercept	6.8053	1.0267	10	6.63	<0.0001
3,5-T2	0.7105	1.1150	171	0.64	0.5249
t	0.004987	0.01708	171	0.29	0.7707
3,5-T2 * t	−0.03422	0.02665	171	−1.28	0.2009

**Table 3:** Changes in 3,5-T2 and 3-T1AM over time in the patients with hypothyroidism treated with LT3.

**Weekly Sampling of 3,5-T2 Concentrations**					
**Effect**	**Estimate**	**Standard Error**	**DF**	***t* Value**	***p* Value**
Intercept	0.9386	0.07048	17	13.32	<0.0001
Week (slope)	−0.01177	0.01731	101	−0.68	0.4982
**Hourly Sampling of 3,5-T2** **Concentrations after the Final LT3 Dose of the Study**					
**Effect**	**Estimate**	**Standard Error**	**DF**	***t* Value**	***p* Value**
Intercept	0.8522	0.1014	17	8.41	<0.0001
Hour (slope)	0.01957	0.01271	160	1.54	0.1256
**Weekly Sampling of 3-T1AM Concentrations**					
**Effect**	**Estimate**	**Standard Error**	**DF**	***t* Value**	***p* Value**
Intercept	5.9772	1.1060	17	5.40	<0.0001
Week (slope)	−0.1769	0.1936	95	−0.91	0.3633
**Hourly Sampling of 3-T1AM Concentrations after the Final LT3 Dose of the Study**					
**Effect**	**Estimate**	**Standard Error**	**DF**	***t* Value**	***p* Value**
Intercept	5.9772	1.1060	17	5.40	<0.0001
Hour (slope)	−0.1769	0.1936	95	−0.91	0.3633

**Table 4:** Correlations between 3,5-T2 and 3-T1AM concentrations during the weekly sampling in the patients with hypothyroidism. (* = interaction term).

**Correlation between 3,5-T2 and 3-T1AM (Model without Using an Interaction Term between 3,5-T2 and Time)**					
**Effect**	**Estimate**	**SE**	**DF**	***t* Value**	***p* Value**
Intercept	4.7959	1.3822	17	3.47	0.0029
3,5-T2	1.2080	0.8232	92	1.47	0.1457
Week	−0.1289	0.1981	92	−0.65	0.5167
**Correlation between 3,5-T2 and 3-T1AM (Model Using an Interaction Term between 3,5-T2 and Time)**					
**Effect**	**Estimate**	**SE**	**DF**	***t* Value**	***p* Value**
Intercept	4.8386	1.9390	17	2.50	0.0232
3,5-T2	1.1742	1.7042	91	0.69	0.4926
Week	−0.1395	0.4326	91	−0.32	0.7478
3,5-T2 * week	0.01183	0.4371	91	0.03	0.9785

**Table 5:** Correlations between 3,5-T2 and T1AM concentrations during the hourly sampling in the patients with hypothyroidism. (* = interaction term).

**Correlation between 3,5-T2 and 3-T1AM (Model without Using an Interaction Term between 3,5-T2 and Time)**					
**Effect**	**Estimate**	**SE**	**DF**	***t* Value**	***p* Value**
Intercept	3.8733	1.2091	17	3.20	0.0052
3,5-T2	2.4540	0.8401	144	2.92	0.0041
Hour	−0.1623	0.1338	144	−1.21	0.2274
**Correlation between 3,5-T2 and 3-T1AM (Model Using an Interaction Term between 3,5-T2 and Time)**					
**Effect**	**Estimate**	**SE**	**DF**	***t* Value**	***p* Value**
Intercept	3.7716	1.5724	17	2.40	0.0282
3,5-T2	2.5727	1.4400	143	1.79	0.0761
Hour	−0.1376	0.2774	143	−0.50	0.6208
3,5-T2 * hour	−0.02692	0.2647	143	−0.10	0.9191

## Data Availability

The data presented in this study are available in the Supplementary Data.

## Supplementary Materials

The following supporting information can be downloaded at: https://www.mdpi.com/article/10.3390/metabo12060476/s1, Supplementary Material: Study Data.

## Appendix B

**Table A1:** Analysis of 3,5-T2 levels: Healthy volunteers.

Parameter	Effect	Estimate	StdErr	DF	*t* Value	*p* Value
Gender	Intercept	0.462	0.033	10	14.03	<0.0001
Gender	Female	0.076	0.056	10	1.36	0.2025
Gender	Male	0.000	.	.	.	.
Gender	t	0.001	0.001	237	1.06	0.2906
Race	Intercept	0.515	0.035	10	14.55	<0.0001
Race	AA	−0.068	0.054	10	−1.26	0.2355
Race	Cauc	0.000	.	.	.	.
Race	t	0.001	0.001	237	1.03	0.3044
T3	Intercept	0.488	0.042	11	11.73	<0.0001
T3	T3	0.000	0.000	235	−0.02	0.9829
T3	t	0.001	0.001	235	1	0.3173
FT3	Intercept	0.490	0.042	11	11.81	<0.0001
FT3	FT3	−0.001	0.006	235	−0.1	0.9181
FT3	t	0.001	0.001	235	0.99	0.3212
T4	Intercept	0.565	0.090	11	6.28	<0.0001
T4	T4	−0.010	0.011	235	−0.91	0.3613
T4	t	0.001	0.001	235	0.87	0.3853
FT4	Intercept	0.425	0.169	11	2.51	0.0289
FT4	FT4	0.066	0.172	234	0.38	0.7018
FT4	t	0.001	0.001	234	1.07	0.2876
TSH	Intercept	0.491	0.042	11	11.81	<0.0001
TSH	TSH	−0.004	0.030	235	−0.12	0.901
TSH	t	0.001	0.001	235	1.02	0.3092
Age	Intercept	0.663	0.096	10	6.91	<0.0001
Age	Age	−0.006	0.003	10	−1.91	0.0847
Age	t	0.001	0.001	237	1.06	0.2913
BMI	Intercept	0.518	0.147	10	3.51	0.0056
BMI	BMI	−0.001	0.005	10	−0.21	0.8371
BMI	t	0.001	0.001	237	1.01	0.3112

**Table A2:** Analysis of 3-T1AM levels: Healthy volunteers.

Parameter	Effect	Estimate	StdErr	DF	*t* Value	*p* Value
Gender	Intercept	5.6329	0.9869	9	5.71	0.0003
Gender	Female	3.9067	1.5637	9	2.5	0.034
Gender	Male	0	.	.	.	.
Gender	t	−0.01318	0.00927	174	−1.42	0.157
Race	Intercept	7.4497	1.2191	9	6.11	0.0002
Race	AA	−0.6011	1.7481	9	−0.34	0.7389
Race	Cauc	0	.	.	.	.
Race	t	−0.0135	0.00932	174	−1.45	0.1492
3,5-T2	Intercept	7.0819	1.0007	10	7.08	<0.0001
3,5-T2	3,5-T2	0.1785	1.0375	172	0.17	0.8636
3,5-T2	t	−0.01344	0.00932	172	−1.44	0.1511
T3	Intercept	6.8562	0.9705	10	7.06	<0.0001
T3	T3	0.00156	0.00225	172	0.69	0.4881
T3	t	−0.00857	0.00925	172	−0.93	0.3555
FT3	Intercept	6.9264	0.968	10	7.16	<0.0001
FT3	FT3	0.04541	0.08474	172	0.54	0.5927
FT3	t	−0.00876	0.00924	172	−0.95	0.3447
T4	Intercept	7.4602	1.9424	10	3.84	0.0033
T4	T4	−0.04051	0.2198	172	−0.18	0.854
T4	t	−0.00936	0.00935	172	−1	0.3185
FT4	Intercept	10.7803	3.8213	10	2.82	0.0181
FT4	FT4	−3.6306	3.7511	171	−0.97	0.3345
FT4	t	−0.01359	0.01027	171	−1.32	0.1875
TSH	Intercept	6.9995	1.0955	10	6.39	<0.0001
TSH	TSH	0.1292	0.5919	172	0.22	0.8275
TSH	t	−0.00925	0.00927	172	−1	0.3197
Age	Intercept	11.6963	2.7959	9	4.18	0.0024
Age	Age	−0.1551	0.09157	9	−1.69	0.1246
Age	t	−0.0139	0.00931	174	−1.49	0.1371
BMI	Intercept	14.3954	4.146	9	3.47	0.007
BMI	BMI	−0.2682	0.151	9	−1.78	0.1095
BMI	t	−0.01377	0.00933	174	−1.48	0.1417

**Table A3:** Analysis of 3,5-T2 levels: Participants with hypothyroidism, weekly.

Effect	Estimate	StdErr	DF	*t* Value	*p* Value
Intercept	1.184	0.145	16.000	8.18	<0.0001
F	−0.2743	0.142	16.000	−1.93	0.071
M	0	.	.	.	.
w	−0.0121	0.017	101.000	−0.72	0.4761
Intercept	0.8704	0.2092	14	4.16	0.001
AA	0.1752	0.2285	14	0.77	0.4558
Asian	−0.2413	0.276	14	−0.87	0.3966
Caus	0.07069	0.2075	14	0.34	0.7384
Hisp	0	.	.	.	.
w	−0.0115	0.0172	101	−0.67	0.5067
Intercept	0.9133	0.126	17.000	7.25	<0.0001
T3	0.00025	0.001	99.000	0.24	0.8132
w	−0.0118	0.017	99.000	−0.68	0.4987
Intercept	0.8757	0.182	17.000	4.82	0.0002
FT3	0.02262	0.061	100.000	0.37	0.7104
w	−0.0111	0.017	100.000	−0.64	0.5241
Intercept	0.6654	0.117	17.000	5.67	<0.0001
T4	0.03276	0.012	100.000	2.76	0.007
w	0.01879	0.019	100.000	0.96	0.3371
Intercept	0.6944	0.131	17.000	5.31	<0.0001
FT4	0.2402	0.113	100.000	2.12	0.0365
w	0.01592	0.021	100.000	0.77	0.4431
Intercept	0.9254	0.073	17.000	12.69	<0.0001
TSH	0.00502	0.006	100.000	0.84	0.4023
w	−0.0132	0.017	100.000	−0.75	0.4526
Intercept	0.9549	0.196	16.000	4.88	0.0002
Age	−0.0004	0.005	16.000	−0.08	0.9335
w	−0.012	0.018	101.000	−0.68	0.4959
Intercept	0.9549	0.196	16.000	4.88	0.0002
Age	−0.0004	0.005	16.000	−0.08	0.9335
w	−0.012	0.018	101.000	−0.68	0.4959

**Table A4:** Analysis of 3-T1AM levels: Participants with hypothyroidism, weekly.

Effect	Estimate	StdErr	DF	*t* Value	*p* Value
Intercept	3.8498	2.932	16.000	1.31	0.2077
F	3.4714	3.009	16.000	1.15	0.2655
M	0	.	.	.	.
w	−0.2391	0.200	95.000	−1.2	0.2341
Intercept	7.4535	4.1736	14	1.79	0.0958
AA	1.761	4.7221	14	0.37	0.7148
Asian	−2.3088	5.7481	14	−0.4	0.694
Caus	−0.879	4.265	14	−0.21	0.8397
Hisp	0	.	.	.	.
w	−0.2463	0.2022	95	−1.22	0.2263
Intercept	5.8185	1.381	17.000	4.21	0.0006
3,5-T2	1.3062	0.873	92.000	1.5	0.1378
w	−0.2389	0.202	92.000	−1.18	0.241
Intercept	6.1262	1.482	17.000	4.13	0.0007
T3	0.00875	0.010	93.000	0.87	0.387
w	−0.2402	0.200	93.000	−1.2	0.2325
Intercept	7.4801	1.937	17.000	3.86	0.0012
FT3	−0.1758	0.571	94.000	−0.31	0.7587
w	−0.2508	0.201	94.000	−1.25	0.2159
Intercept	6.9359	1.631	17.000	4.25	0.0005
T4	0.00591	0.135	94.000	0.04	0.965
w	−0.2402	0.239	94.000	−1	0.3181
Intercept	4.457	1.716	17.000	2.6	0.0188
FT4	2.2835	1.174	94.000	1.95	0.0547
w	0.03499	0.245	94.000	0.14	0.8869
Intercept	6.9543	1.094	17.000	6.36	<0.0001
TSH	0.01867	0.051	94.000	0.37	0.7131
w	−0.2531	0.201	94.000	−1.26	0.212
Intercept	6.8283	3.806	16.000	1.79	0.0917
Age	0.0042	0.096	16.000	0.04	0.9655
w	−0.246	0.201	95.000	−1.22	0.2245
Intercept	6.8283	3.806	16.000	1.79	0.0917
Age	0.0042	0.096	16.000	0.04	0.9655
w	−0.246	0.201	95.000	−1.22	0.2245

**Table A5:** Analysis of 3,5-T2 levels: Participants with hypothyroidism, hourly.

	Effect	Estimate	StdErr	DF	*t* Value	*p* Value
Sex	Intercept	1.134	0.269	16	4.21	0.0007
Sex	F	−0.317	0.281	16	−1.13	0.2761
Sex	M	0.000	.	.	.	.
Sex	t	0.020	0.013	160	1.54	0.1253
Race	Intercept	0.475	0.3856	14	1.23	0.2383
Race	AA	0.4527	0.4415	14	1.03	0.3226
Race	Asian	0.0209	0.5408	14	0.04	0.9697
Race	Caus	0.4161	0.3968	14	1.05	0.3121
Race	Hisp	0	.	.	.	.
Race	t	0.01958	0.01277	160	1.53	0.1271
T3	Intercept	0.835	0.106	17	7.91	<0.0001
T3	T3	0.000	0.000	159	0.61	0.5457
T3	t	0.019	0.013	159	1.51	0.1337
FT3	Intercept	0.844	0.109	17	7.77	<0.0001
FT3	FT3	0.002	0.011	159	0.21	0.8321
FT3	t	0.019	0.013	159	1.52	0.1295
T4	Intercept	0.795	0.112	17	7.12	<0.0001
T4	T4	0.016	0.014	159	1.12	0.266
T4	t	0.020	0.013	159	1.57	0.1173
FT4	Intercept	0.659	0.155	17	4.27	0.0005
FT4	FT4	0.435	0.267	159	1.63	0.1049
FT4	t	0.020	0.013	159	1.56	0.1219
TSH	Intercept	0.803	0.126	17	6.39	<0.0001
TSH	TSH	0.013	0.019	159	0.69	0.4921
TSH	t	0.021	0.013	159	1.6	0.1121
Age	Intercept	0.532	0.359	16	1.48	0.1573
Age	Age	0.008	0.009	16	0.93	0.3662
Age	t	0.020	0.013	160	1.54	0.1257
BMI	Intercept	0.532	0.359	16	1.48	0.1573
BMI	Age	0.008	0.009	16	0.93	0.3662
BMI	t	0.020	0.013	160	1.54	0.1257

**Table A6:** Analysis of 3-T1AM levels: Participants with hypothyroidism, hourly.

	Effect	Estimate	StdErr	DF	*t* Value	*p* Value
Sex	Intercept	4.330	2.680	16	1.62	0.1258
Sex	F	1.782	2.782	16	0.64	0.531
Sex	M	0.000	.	.	.	.
Sex	t	−0.110	0.136	145	−0.81	0.4217
Race	Intercept	4.7631	3.4473	14	1.38	0.1887
Race	AA	4.752	3.9352	14	1.21	0.2472
Race	Asian	−3.0023	4.9113	14	−0.61	0.5508
Race	Caus	0.7309	3.5387	14	0.21	0.8393
Race	Hisp	0	.	.	.	.
Race	t	−0.1148	0.1339	145	−0.86	0.3926
3,5-T2	Intercept	3.873	1.209	17	3.2	0.0052
3,5-T2	3,5-T2	2.454	0.840	144	2.92	0.0041
3,5-T2	t	−0.162	0.134	144	−1.21	0.2274
T3	Intercept	6.561	1.063	17	6.17	<0.0001
T3	T3	−0.004	0.002	144	−2	0.0473
T3	t	−0.100	0.135	144	−0.74	0.4591
FT3	Intercept	6.511	1.102	17	5.91	<0.0001
FT3	FT3	−0.161	0.117	144	−1.38	0.1712
FT3	t	−0.102	0.136	144	−0.75	0.4515
T4	Intercept	6.315	1.156	17	5.46	<0.0001
T4	T4	−0.112	0.154	144	−0.72	0.4703
T4	t	−0.113	0.136	144	−0.83	0.4099
FT4	Intercept	6.406	1.586	17	4.04	0.0009
FT4	FT4	−1.132	2.796	144	−0.41	0.6861
FT4	t	−0.109	0.137	144	−0.8	0.4245
TSH	Intercept	5.168	1.196	17	4.32	0.0005
TSH	TSH	0.196	0.182	144	1.08	0.2821
TSH	t	−0.095	0.135	144	−0.7	0.4849
Age	Intercept	4.186	3.577	16	1.17	0.2591
Age	Age	0.045	0.090	16	0.51	0.6204
Age	t	−0.109	0.136	145	−0.8	0.4246
BMI	Intercept	4.186	3.577	16	1.17	0.2591
BMI	Age	0.045	0.090	16	0.51	0.6204
BMI	t	−0.109	0.136	145	−0.8	0.4246
