# Supplementary material for: Changes in Thyroid Metabolites after Liothyronine Administration: A Secondary Analysis of Two Clinical Trials That Incorporated Pharmacokinetic Data
Source: Metabolites. 2022 May 24;12(6):476. doi: 10.3390/metabo12060476 (PMC9227779; doi:10.3390/metabo12060476)
Supplement: Supplementary file 1 [file metabolites-12-00476-s001.zip › metabolites-1708267-supplementary.pdf]

Supplementary Material: Study Data

patient	time_Pk	treatment	TSH	T4	T3	FT4
**1**	0	30	12	3.1	84.3	0.43
**2**	0	30	0.566	8.1	184.6	0.92
**3**	0	30	3.4	3.5	69.4	0.68
**4**	0	30	2.42	7.2	97.8	1.03
**5**	0	30	1.8	11.1	156.2	0.84
**6**	0	30	7.21	1.1	59.6	0.29
**7**	0	30	11.2	0.9	78.3	0.19
**8**	0	30	4.22	1.1	84.9	0.28
**9**	0	30	4.8	2.5	86.6	0.27
**10**	0	30	1.22	4.7	75.1	0.41
**11**	0	30	1.27	3.4	104	0.69
**12**	0	30	0.153	1.3	103.7	0.21
**13**	0	30	3.93	7.9	124.8	0.82
**14**	0	30	2.07	1.3	98.6	0.16
**15**	0	30	0.038	1.3	104.8	0.1
**16**	0	30	8.93	0.5	59.6	0.1
**17**	0	30	2.45	4.9	83.6	0.45
**18**	0	30	1.52	1.7	74.6	0.12
**1**	0.5	30	12.5	3.9	85.6	0.44
**2**	0.5	30	0.562	7.4	205.6	0.89
**3**	0.5	30	3.09	3.6	76.1	0.68
**4**	0.5	30	2.02	7.2	147	1
**5**	0.5	30	1.47	10.1	248.8	0.83
**6**	0.5	30	6.88	1.6	93.8	0.27
**7**	0.5	30	12.2	1.1	197.4	0.22
**8**	0.5	30	3.71	3.2	101.2	0.28
**9**	0.5	30	3.87	2.6	108.8	0.27
**10**	0.5	30	1.16	3.3	99.1	0.46
**11**	0.5	30	1.3	5.7	138.3	0.73
**12**	0.5	30	0.126	1.6	93	0.21
**13**	0.5	30	3.78	7.2	125.8	0.82
**14**	0.5	30	2.05	0.6	100.8	0.14
**15**	0.5	30	0.037	0.5	353.7	0.13
**16**	0.5	30	8.22	1.3	49.9	0.13
**17**	0.5	30	2.4	2.8	90.6	0.45
**18**	0.5	30	1.53	1.6	89.5	0.15
**1**	1	30	11.6	3.6	131.8	0.42
**2**	1	30	0.515	7.6	300.2	0.89
**3**	1	30	3.1	3.2	99.4	0.69
**4**	1	30	1.84	6.9	228.9	0.95
**5**	1	30	1.39	10.9	335.8	0.82
**6**	1	30	7.2	1.4	123.4	0.29
**7**	1	30	11.5	1.5	353.7	0.19

**8**	1	30	4.31	0.5	286	0.29
**9**	1	30	4.03	2.7	261.4	0.26
**10**	1	30	1.03	2.6	217.8	0.44
**11**	1	30	1.35	4	145.8	0.69
**12**	1	30	0.133	1.9	291	0.19
**13**	1	30	3.33	7.6	120.7	0.82
**14**	1	30	2.1	0.7	198	0.15

FT3	Sex	Age	Race	Etiology	Weight.(kg)	T1_AM
2.31	F	25	Hisp	Hashimotos	81.9	8.07136034
3.32	F	24	Caus	Hashimotos	65	0
2.88	F	35	Asian	Hashimotos	55.5	2.05257663
3.52	M	43	Caus	Hashimotos	94.7	0
3.46	F	24	Caus	Hashimotos	68.3	11.8305297
2.33	F	45	Caus	Hashimotos	81	7.14874336
2.36	F	56	AA	Hashimotos	66.5	14.8543153
2.5	F	31	Caus	Hashimotos	79.6	10.8070192
2.02	F	40	Caus	Hashimotos	78.9	4.61795023
2.28	F	41	Caus	Hashimotos	69.2	4.72057306
3.29	F	32	AA	Hashimotos	131.2	3.10361757
3.84	F	24	Caus	Hashimotos	100.2	9.54904128
2.98	F	35	Caus	Hashimotos	88.3	7.03477395
2.4	F	47	Caus	Hashimotos	119	1.72152859
3	F	47	Caus	Hashimotos	52.8	6.61313177
1.7	M	50	AA	ThyX	96.9	3.77064303
2.3	F	50	Caus	Hashimotos	68.3	1.82742244
1.62	F	36	Caus	Hashimotos	74.5	1.03980753
2.66	F	25	Hisp	Hashimotos	81.9	6.25789184
3.56	F	24	Caus	Hashimotos	65	0
2.87	F	35	Asian	Hashimotos	55.5	2.5673881
4.49	M	43	Caus	Hashimotos	94.7	4.69178393
4.76	F	24	Caus	Hashimotos	68.3	12.9135444
3.18	F	45	Caus	Hashimotos	81	7.25949636
5.46	F	56	AA	Hashimotos	66.5	16.8726483
2.81	F	31	Caus	Hashimotos	79.6	9.96270164
2.79	F	40	Caus	Hashimotos	78.9	7.55671632
2.9	F	41	Caus	Hashimotos	69.2	5.94118803
4.21	F	32	AA	Hashimotos	131.2	1.62969621
2.53	F	24	Caus	Hashimotos	100.2	7.35272174
3	F	35	Caus	Hashimotos	88.3	9.71674299
2.34	F	47	Caus	Hashimotos	119	1.94470364
8.47	F	47	Caus	Hashimotos	52.8	4.76984686
1.76	M	50	AA	ThyX	96.9	1.49512246
2.41	F	50	Caus	Hashimotos	68.3	5.65824291
2.17	F	36	Caus	Hashimotos	74.5	1.98040237
3.64	F	25	Hisp	Hashimotos	81.9	6.37173767
5.16	F	24	Caus	Hashimotos	65	0

4.33	F	35	Asian	Hashimotos	55.5	1.60571887
6.38	M	43	Caus	Hashimotos	94.7	4.84052615
6.43	F	24	Caus	Hashimotos	68.3	12.7037188
4.36	F	45	Caus	Hashimotos	81	5.79574924
8.82	F	56	AA	Hashimotos	66.5	15.7742356
6.55	F	31	Caus	Hashimotos	79.6	9.95322233
5.06	F	40	Caus	Hashimotos	78.9	7.47028912
5.49	F	41	Caus	Hashimotos	69.2	4.28569242
4.41	F	32	AA	Hashimotos	131.2	3.1610294
4.97	F	24	Caus	Hashimotos	100.2	0
2.95	F	35	Caus	Hashimotos	88.3	10.4170504
4.03	F	47	Caus	Hashimotos	119	1.3029281

T2

0.50198679

0.56520977

0.44287

2.02524558

1.28503976

0.77481922

1.1119973

0.92968295

0.97842302

0.96491222

1.03656728

0.47241755

1.01006774

0.68038307

0.49890778

0.66406372

0.75995792

0.8809594

0.45944339

0.97126974

0.53094089

1.50198012

1.40575902

0.55375611

1.29038297

0.98671801

0.91446243

0.87154085

1.0742467

0.458561

0.8645137
0.6419527
0.44483844
0.70199974
0.82275997
0.96759049
0.43192389
0.71550904
0.51989919
1.60479992
1.35625751
0.57448796
1.25705413
0.82789053
0.83228586
0.94115366
0.95412035
0
0.85375398
0.66646274
0.43654645
0.58965868
0.8600566
1.01856874
0.44883528
0.63214454
0.50618391
1.30018503
1.19724195
0.58006603
1.49603033
0.79345833
1.04174679
1.39917832
1.04576082
0.44980116
0.79812628
0.77129909
0.45485123
0.541323
1.35923771
1.19623283
0.41140404

0.61997444
0.48461255
1.31417018
1.27304666
0.51096747
1.42805685
0.7898742
0.79212155
1.61086152
1.03819605
0.35758272
0.68785068
0.94664705
0.30263177
0.4595843
1.23911095
1.49926132
0.36692351
0.63388769
0.55852046
1.64458862
0.69658261
0.49734653
1.85186899
0.83796341
1.07712915
1.46471458
1.02353394
0.24559582
0.68902984
0.98406663
0.36820818
0.49655934
1.01279294
2.03979321
0.45529948
0.6946822
0.43808355
1.74401822
1.08404694
0.49296273
2.00209937
0.93872424

1.26756087
1.40711564
0.95401885
0.4147179
0.81065065
0.99774814
0.65220661
0.50799675
0.97124685
2.03289603
0.22385334
0.70490879
0.45487103
1.72812633
1.25915645
0.38771422
1.76139418
1.12059185
1.18263785
1.42466666
0.86849944
0.35486372
0.72188888
1.00854412
0.55403835
0.50293556
0.89966883
2.103292
0.2770099
0.7391173
0.51075421
2.20175897
1.38299224
0.42229486
1.79780305
0.9804577
1.45159652
1.51690159
0.92481239
0.37619405
0.71950104
0.93496507
0.69516609

0.48653064
0.98981904
1.60352744
0.69940351
0.76031882
0.74413412
1.828905
1.30328654
0.39960994
1.64153598
0.86797968
1.71797919
1.3191878
1.02380693
0.38166567
0.66029897
0.90919483
0.63608564
0.47610476
0.975803
1.64246375
